# Calibration of Electrochemical Sensors for Nitrogen Dioxide Gas Detection Using Unmanned Aerial Vehicles

**DOI:** 10.3390/s20247332

**Published:** 2020-12-20

**Authors:** Raphael Mawrence, Sandra Munniks, João Valente

**Affiliations:** 1 Laboratory of Geo-Information Sciences and Remote Sensing at Wageningen University & Research (WUR), Droevendaalsesteeg 3, 6708 PB Wageningen, The Netherlands; mawrenceraphael@gmail.com; 2 Wageningen Food Safety Research, Akkermaalsbos 2, 6708 WB Wageningen, The Netherlands; Sandra.munniks@wur.nl; 3 Information Technology Group, Wageningen University, Hollandseweg 1, 6706 KN Wageningen, The Netherlands

**Keywords:** air quality monitoring network, calibration, electrochemical sensor, nitrogen dioxide, spatial resolution, UAV, unmanned aerial vehicle

## Abstract

For years, urban air quality networks have been set up by private organizations and governments to monitor toxic gases like NO_2_. However, these networks can be very expensive to maintain, so their distribution is usually widely spaced, leaving gaps in the spatial resolution of the resulting air quality data. Recently, electrochemical sensors and their integration with unmanned aerial vehicles (UAVs) have attempted to fill these gaps through various experiments, none of which have considered the influence of a UAV when calibrating the sensors. Accordingly, this research attempts to improve the reliability of NO_2_ measurements detected from electrochemical sensors while on board an UAV by introducing rotor speed as part of the calibration model. This is done using a DJI Matrice 100 quadcopter and Alphasense sensors, which are calibrated using regression calculations in different environments. This produces a predictive r-squared up to 0.97. The sensors are then calibrated with rotor speed as an additional variable while on board the UAV and flown in a series of flights to evaluate the performance of the model, which produces a predictive r-squared up to 0.80. This methodological approach can be used to obtain more reliable NO_2_ measurements in future outdoor experiments that include electrochemical sensor integration with UAV’s.

## 1. Introduction

**Figure 1:**
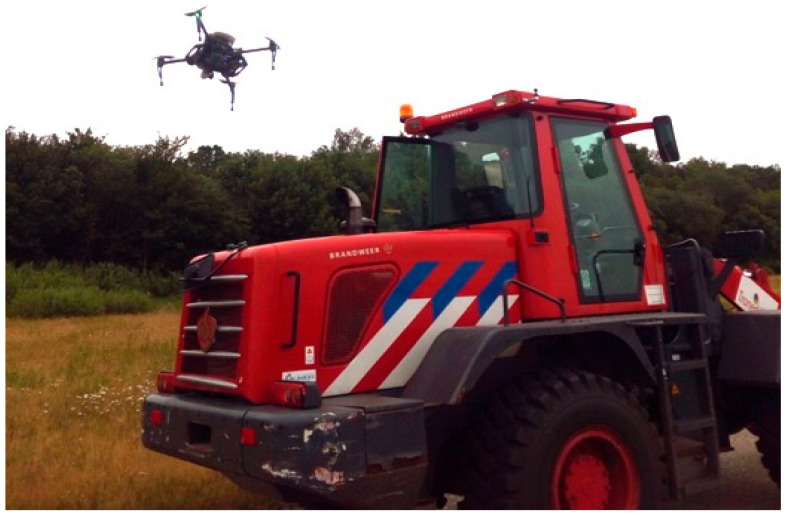
The DJI Matrice 100 hovers above a 2005 Foton tractor at the Twente Safety Campus in Enschede, The Netherlands.

Current air quality networks are meticulously distributed across the planet, which is determined by different land-use characteristics. However, due to their high cost and need for routine maintenance, the distribution of these networks can be widely spaced [1]. There are interpolation methods that have been tested to estimate the concentrations of pollutants away from such stations, though research suggests that results of these techniques are still affected by the inherent spatial distribution of the stations and the temporal resolution of the data [2]. This is a spatial concern. While the following research is in its infancy, electrochemical sensors and their compact design, relatively low power usage, affordability, accuracy, and reliability allows for their integration with Unmanned Aerial Vehicles (UAVs), which offers a unique opportunity to measure toxic gases like nitrogen dioxide NO_2_—one of the most harmful gases to human health—in a wide range of environments for which we do not have air quality information [3,4,5,6,7,8,9,10,11,12,13,14]. Accordingly, integrating electrochemical sensors with UAVs may help solve the spatial concern of toxic gas detection by delivering useful insights about local air quality and complimenting data collected under passive sampling techniques by measurement stations within air quality networks (Figure 1).

Despite their proven strengths, sensor readings from electrochemical sensors are vulnerable to the cross sensitivity of ozone and exogenous variables like temperature, relative humidity, and even wind, which can lead to concentration errors when functioning outside of the suggested operational rage but can be accounted for throughout the system’s calibration process [5,7,10]. This process begins by collecting data from a ground truth or reference measurement that takes place at the same location and time that the sensor is collecting data, which can be in an indoor or outdoor setting. An outdoor calibration typically refers to placing the sensor that needs to be calibrated next to an air quality station, which measures the same gas of interest for a predetermined amount of time [5]. For an indoor calibration, compressed air systems can be used with meters, regulators, and pumps that flow into and out of a controlled container holding the sensor [4,7,8,9]. Sensors also experience what is known as drift, which refers to the amount of time that passes before they need to be calibrated again. However, drift has a so-called “second-order” effect on a sensors sensitivity, whereas temperature (T) or relative humidity (RH) have “first-order” effects on sensitivity [4,15]. This means that temperature and relative humidity are more important to consider when optimizing a calibration for electrochemical sensors. After the data has been collected by both the ground truth sensor and the sensor that needs to be calibrated, an algorithm can then be used to account for environmental characteristics that might be interfering with the sensor reading. Various models have been applied to validate the data such as using regressions, artificial neural networks, or other non-linear algorithms and supervised learning techniques [3,5,16,17,18,19].

Depending on the accuracy desired, sensors are calibrated using the manufacturer’s factory-supplied constants. However, as Mijling et al. demonstrated using electrochemical sensors developed by Alphasense (Essex, UK), this does not yield the most reliable results as these constants do not always account for all external factors that influence sensor readings [5]. In order to account for these effects, Mijling et al. proposed calibration models that differ from the manufacturer’s recommended calibration method, each containing a linear combination of the raw sensor reading with an additional variable that accounts for either T, RH, ozone (O_3_), or all [5]. Their research determined that the ranges of the coefficient of determination (R^2^) increases when performing a linear combination of all variables (T, RH, and O_3_) from 0.3–0.7 (when no combination was performed) to 0.6–0.9. The Mijling et al. results provide a direction to which this study can calibrate electrochemical sensors while on board an UAV [5].

While there are different ways to calibrate sensors ensuring their accuracy and reliability, future experiments have yet to offer a new calibration procedure that more appropriately reflects their integration with UAV’s [5]. Current research that pertains to electrochemical sensor-UAV integration for the detection of toxic gases like NO_2_ use traditional methods for sensor calibration, which may result in unrealistic concentrations of the target gas. It is thus expected that including an additional variable in Mijling et al.’s calibration model that may influence electrochemical gas detection using UAVs, such as rotor speed, would produce more reliable results than traditional calibration methods [5]. Therefore, the aim of the research is to develop a unique calibration model for electrochemical sensors and their integration with UAV’s to help improve the reliability of data collected, which could ultimately expand the resolution of and capacity for detecting toxic gases along air quality monitoring networks.

## 2. Methodology for Calibrating Electrochemical Sensors for NO_2_ Gas Detection on Board UAV’s

The methodology of calibrating electrochemical sensors for NO_2_ gas detection and UAV integration is achieved in three phases. Phase 1 uses traditional calibration conditions at an official station outdoors; Phase 2 calibrates the sensors at an official station indoors, and Phase 3 calibrates the sensors outdoors using an UAV. For Phase 1, the sensor is calibrated to data derived from the Amsterdam Vondelpark measuring station, which is part of the Public Health Service of Amsterdam (GGD Amsterdam). In Phase 2, the sensor is calibrated to the known concentrations of an indoor gas source at the GGD Amsterdam facility. Both Phases 1 and 2 use different calibration models founded on the calibration procedure proposed by Mijling et al., which is a linear combination of the raw sensor reading, T, and RH [5]. For Phase 3, each electrochemical sensor is then calibrated with the best performing calibration model from Phase 1 and Phase 2, which is determined by specific standards outlined in Section 2.4. This final phase is split into two stages. Stage 1 calibrates the sensors with an UAV, and Stage 2 validates the calibration through an experiment where the UAV is flown along a flight path dependent on the wind direction near an outdoor emission source. Data analysis methods found in the third subsection determines if rotor speed has any significant effect on sensor readings while mounted on an UAV. Careful filtering of raw data and startup time are considered for each phase. All three phases use a simple multivariable regression (MR) in order to derive coefficients that are applied to the linear combination of variables [5]. Phases 1 and 2 use polynomial and logarithmic functions to better account for possible non-linear changes in the sensor reading.

### 2.1. Materials and Instrumentation

**Figure 2:**
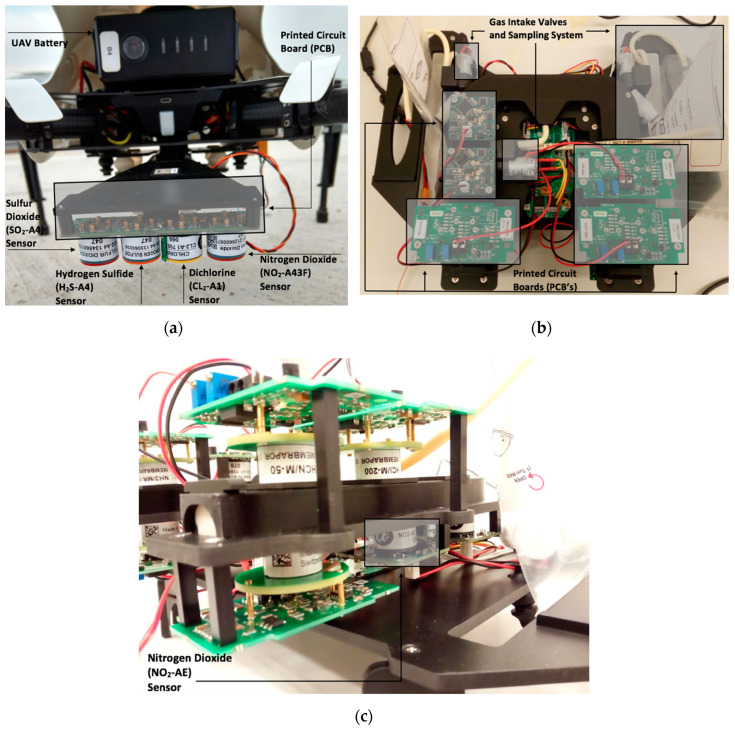
(**a**) UAV mount for the NO_2_-A43F system setup; (**b**) top of UAV mount for the NO_2_-AE setup; (**c**) side of UAV mount for the NO_2_-AE setup.

Traditional electrochemical sensors operate by chemical reactions taking place inside the sensor chamber after the gas of interest passes through the working electrode (WE), reference electrode, and counter electrode, which generates electrical signals—in volts—proportional to the gas concentration [4]. Other electrochemical sensors utilize a fourth electrode known as the auxiliary electrode (AE), which attempts to balance background currents that tend to interfere with low-range sensor readings [4,20]. Similar to the sensor used in the study of Mijling et al., the Alphasense NO_2_-A43F sensor is a four-electrode system used specifically for low gas concentrations (Figure 2a), and Alphasense’s NO_2_-AE sensor is a standard three-electrode system developed for high gas concentrations (Figure 2b,c). Using both can provide useful insight into which type of sensor is better for detecting NO_2_ while attached to an UAV. Both are used throughout Phases 1, 2, and 3 of this research and are mounted on plastic structures designed by the Dutch company Robor Electronics B.V. (Bentelo, The Netherlands), which can attach directly below the UAV (Figure 2) [21]. The raw sensor reading for the NO_2_-A43F sensor is therefore the WE and AE, whereas the sensor reading for the NO_2_-AE sensor is only the WE. A potentiostatic circuit regulates the raw sensor measurements, which are generated in counts. The NO_2_-A43F sensor has a sensitivity of −175 to −500 nA/ppm at 2 ppm NO_2_ and a response time of less than 60 s at t_90_ from zero to 2 ppm NO_2_. For the NO_2_-AE sensor, it has a sensitivity of −70 to −170 nA/ppm at 10 ppm NO_2_ and a response time of less than 40 s at t_90_ from zero to 10 ppm NO_2_. In terms of stability, the NO_2_-AE sensor has an operational life of two years until only 80% to the original signal is retained, whereas the NO_2_-A43F sensor has 50% retention after the same period. The NO_2_-A43F sensor in the following experiments is on its second year of operation, whereas the NO_2_-AE is on its first year. Other sensors are also included on the plastic mounts but are not used for this research. They typically operate between 5–30 °C and have a warmup time of about two hours, meaning that any recordings outside of these ranges are filtered. Filtering data this way is a standard procedure [5]. Furthermore, data is recorded approximately every two seconds. Further information such as sensor configurations can be found on the technical specifications sheet online [22,23].

Both sensors use the Long-Range BT FLIR LCD touch remote controller for wireless data acquisition at a coverage of 2 km [24]. The NO_2_-AE sensor also uses an antenna to enhance the distance cover, which is not needed for the NO_2_-A43F sensor. Each sensor utilizes a separate software for data collection. The NO_2_-A43F sensor employs the RealTerm: Serial/Transmission Control Protocol Terminal Capture Program version 2.0.0.70 for instrument control and data logging, whereas the NO_2_-AE sensor uses StrokeReader ActiveX [25,26].

For Phase 3, the DJI Matrice 100 used (DJI, Shenzhen, China) is a UAV system that weighs approximately 2400 g with a max takeoff weight of 3600 g. The unit also comes equipped with a wireless communication network that can be connected and flown via a computer with a maximum unobstructed transmission distance of 5 km. During this phase, a 2005 Foton FL936F tractor (Shandong Foton Heavy Industries Co., Ltd, Shandong, China) operates as the emission source, which is supplied by the Twente Safety Campus in Enschede, The Netherlands—a facility that provides training simulations for the fire department, police, defense, and medical assistance [27]. It is expected that this machine’s exhaust produces a stable emission source to capture NO_2_.

### 2.2. Experiment Setup

**Figure 3:**
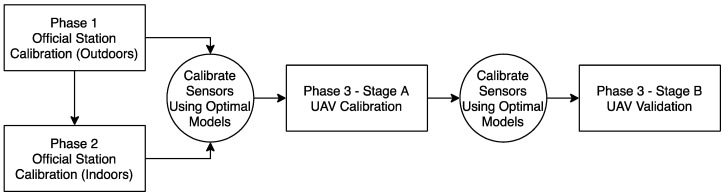
Experimental setup for calibrating electrochemical sensors with UAV integration in.

Three phases:

(1) Phase 1: On 26 November 2018 both sensors were placed on the rooftop of the Amsterdam Vondelpark air quality monitoring station and were left there for a total calibration duration of eight days. They were positioned next to the stations’ sensors that measure NO_2_ and underneath a designed housing so that they would not be exposed to rain or other debris during the calibration process. For unknown reasons, the NO_2_-A43F sensor stopped recording after 19 h. As a result of having too few observations, this sensor is not calibrated during this Phase. A data split for the NO_2_-AE sensor occurs at approximately 48-h intervals between the start and end time.
(2) Phase 2: Approximately two hours after Phase 1 ended, the sensors were taken to GGD Amsterdam’s indoor facility to begin Phase 2. Both sensors were exposed to different gases at different concentrations starting on 4 December 2018 at 13:00 and ending at 10:13 on 12 December 2018, which was controlled and monitored by the Environment S.A AC32 E-Series particulate analyzer – a micro-sensor designed specifically for controlling NO_x_ gases. The sensors were left on throughout this entire duration. The period at which the sensors were exposed only to NO_2_ was from 10:35–12:00 (85 min) on 11 December 2018. Startup time is therefore not an issue during this phase. The data is split at approximately 21-min intervals between the start and end time. For this calibration, ground truth readings are 0.045 ppm from 10:35–11:24 and 0.06 from 11:24–12:00. T and RH are not controlled by the micro-sensor and are instead managed by the air-tight conditions of the testing room, which results in consistent readings of T and RH throughout the entire calibration period.
(3) Phase 3: On 16 July 2019, the sensors were taken to the Twente Safety Campus in Enschede, The Netherlands. It was necessary to travel to this location in order to use an emission source capable of releasing enough gas for the sensors to detect. This phase is broken down into two stages: Stage A, which was inspired by previous research activities to calibrate the sensors, and Stage B, which is meant for validating the calibration [28]. The NO_2_-A43F and NO_2_-AE sensors both go through the same Stage A-Stage B processes. A complete overview of Phases 1, 2 and 3 are represented in Figure 3 below.

**Figure 4:**
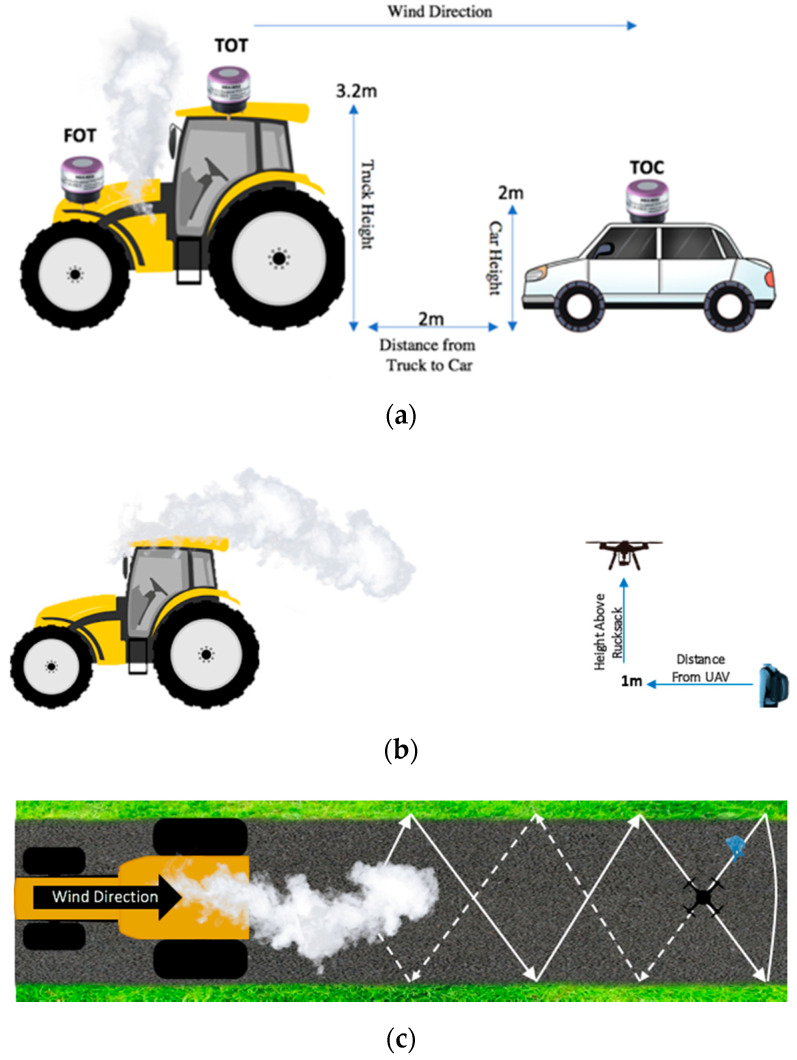
(**a**) The experimental setup for Stage A, where three ground truth locations (purple sensor) are placed on the front of the truck next to the exhaust, on top of the truck behind the exhaust, and two meters away from the truck at a height of two meters on top of a second vehicle; (**b**) Side view of the experimental setup for Stage B. One sensor was attached to the UAV as it flew along a predetermined zig-zag flight path with and against the wind direction. The other sensor was attached to a rucksack and walked approximately one meter below and one meter behind the UAV as it followed the flight path, which was used as a ground truth to validate the calibration of the sensor that was attached to the UAV. This representation is not to scale; (**c**) Top view of the experimental setup for Stage B.

In preparation for Stage A, the calibrated sensors were placed at three stationary positions next to the emission source for approximately 10-min, thus deriving concentrations that are recorded as readings considered to be the ground truth (Figure 4a). Each of these locations were separated into three distinct data acquisition periods, which is illustrated in Figure 4. One location is on the front of the truck next to the exhaust pipe (FOT), another location is on top of the truck behind the exhaust (TOT), and the third is on another vehicle 2 m behind the truck at an elevation of 2 m in the wind direction where the plume is expected to travel (TOC). The ground truth concentration is obtained by taking the coefficients derived from the best performing model from the previous calibration phases and applying them to the raw readings of the sensors at each of these locations. This provides a calibrated NO_2_ ground truth concentration in ppm for Stage A.

Each sensor was then attached to the UAV and flown at each of these three locations measuring the same emission source. The plume from the emission source is assumed to remain a consistent size as the throttle was at the same position during the entire experiment. Due to safety and concerns about damaging the UAV, sensors, and sensor housings, it was not possible to turn the UAV on at each of these three exact locations. Therefore, the UAV flew approximately 1 m away from where the ground truth sensors were located with the exception of the third location where the car could be moved.

Stage B required the UAV to fly in a zigzag pattern with each of the NO_2_ sensors. This flight pattern was utilized so that the sensors could measure NO_2_ over a wider range of the plume by flying with and against the wind direction [29]. As the UAV was flying with one of the sensors, the other sensor was attached to a rucksack and walked along the same flight path at approximately the same altitude as the UAV (Figure 4b,c). The data gathered from the sensor attached to the rucksack were used as ground truth concentrations in order to validate the calibration of the sensor that was attached to the UAV. The same flight and walk pattern was completed twice for each sensor along the exact same path in order to account for any errors during data collection and to optimize the reliability of results.

### 2.3. Data Filtering

Filtering data removed the first two hours of the outdoor and UAV calibrations from Phase 1 and Phase 3, which reflected the startup time for the sensors. The datasets were then split in order to minimize lingering effects from external factors that might be influencing the sensor reading. Calibrations for Phase 1 and Phase 2 split the whole dataset into four equal quarter sections. Despite both sensors being placed at three different locations for Phase 3, it was also necessary to split each of these locations into three different data acquisition periods. This required filtering the NO_2_-A43F and NO_2_-AE sensor data derived from the UAV flight log’s start and stop time. For each of the three phases, sensor readings that were 1.5 interquartile ranges (IQRs) below the first quartile of the readings or 1.5 IQRs above the third quartile of the readings were considered as outliers and eliminated from the dataset. This IQR is used to reflect the 1.5 IQR Rule developed by statistician pioneer John Tukey [30]. Data was then averaged by one hour to reflect the ground truth in Phase 1, and ten seconds to reflect the ground truth in Phase 2. It was not necessary to average the data in Phase 3 despite sensors having identical data collection time. Once all datasets were filtered and averaged accordingly, the best performing calibration model could be applied and more reliable concentrations of NO_2_ can be determined for each phase.

### 2.4. Modelling

Once sensor data has been collected, filtered, and averaged according to specific parameters discussed in the last segment of this section, sensor readings can be included in a MR model where the dependent variable is the ground truth data and the independent variables are the sensor reading (AE and WE in counts), T (in °C), and RH (in %). In addition to this model, polynomial and logarithmic regression models are also used in order to account for potential non-linear occurrences in sensor readings. The coefficients c_0_, c_1_, c_2_, and c_3_ are derived from the best performing model and can then be used to convert raw sensor readings into parts-per-million (ppm) concentrations in Equations (1) and (2):

NO_2_-A43F [ppm] = c_o_ + (c_1_ · WE) + (c_2_ · AE) + (c_3_ · T) + (c_4_ · RH), (1)

NO_2_-AE [ppm] = c_o_ + (c_1_ · WE) + (c_2_ · T) + (c_3_ · RH) (2)

As shown by Mijling et al., more reliable NO_2_ concentrations can be derived from a linear combination of the NO_2_ signal, T, and RH using Equation (1) for the NO_2_-A43F sensor and Equation (2) for the NO_2_-AE sensor [5]. The equations presented here are assuming that T and RH are statistically significant additions to the best performing model during Phase 1 and Phase 2 calibrations, though as stated before it is possible that this may not be the case. Therefore, Equations (1) and (2) may change by removing T, RH, or both.

For the polynomial regression model, polynomials are assigned to each variable (sensor readings, T, and RH) in increments of one power. This creates a new variable in the regression model and is done for each variable until or unless a new variable is no longer statistically significant to the model, which is determined by a *p*-value above 0.05 and used as a statistic for each of the three regression models to determine statistical significance. A logarithmic regression applies a logarithm function to each variable under the same parameters as the polynomial regression model. It is likely that not all variables variables are statistically significant and would therefore not be incorporated in the best performing model. These principles are considered throughout all three phases.

In Phase 2, like the previous phase, there is a linear combination of the NO_2_ signal, T, and RH as seen in Equations (1) and (2) to determine more reliable ppm concentrations of NO_2_ after executing the best performing regression models. The results from Phase 1 and Phase 2 determines which environment—outdoor or indoor—and which model—multivariable, polynomial, logarithmic—and with which coefficients are optimal to calibrate the sensors. The coefficient derived from the model with the environment that has the strongest predictive r-squared where all variables are statistically significant additions to the model are used in order to calibrate the sensors for Phase 3.

Despite there being the necessity to measure NO_2_ under different conditions than Phase 1 and Phase 2, a new calibration model must be implemented. Equations (3) and (4) build on Equations (1) and (2) by incorporating another independent variable that is expected to influence sensor readings: UAV rotor speed (RS). Equation (3) pertains to the NO_2_-A43F sensor and Equation (4) pertains to the NO_2_-AE sensor. Higher order polynomials or logarithmic regressions are not used in this phase in order to simplify analysis:

NO_2_-A43F [ppm] = co + (c_1_ · WE) + (c_2_ · AE) + (c_3_ · T) + (c_4_ · RH) + (c_5_ · RS), (3)

NO_2_-AE [ppm] = co + (c_1_ · WE) + (c_2_ · T) + (c_3_ · RH) + (c_4_ · RS) (4)

The best performing model is considered as the one where all variables associated to the model are statistically significant and have the highest predictive r-squared. Rather than using an in-sample measure calculated with the multiple r-squared or adjusted r-squared, a predictive r-squared can be used to prevent over-fitting a model using an out-of-sample measure, which has been used in literature when selecting optimal models [31,32]. In addition to these statistics, the root mean square error (RMSE) is also considered when choosing the best model, which is defined by the error between the predicted and true values of a dataset.

## 3. Results

Specific details on results can be seen in Table 1, which outlines the sensor used in each phase (except for Stage B of Phase 3), what part of the data split was used or where the sensor was placed during Phase 3, the model used to calibrate the sensors, model components, and finally the statistics and metrics associated with each model. Results presented here are when all variables are statistically significant additions within the model. Results in Stage B of Phase 3 are provided in Table 2.

The best performing model for the NO_2_-AE sensor in this Phase 1 is the MR that includes the sensor reading and RH on the third data split, which lasted between 30 November 2018 at 07:00:00 and 2 December 2018 at 08:00:00. T was not included in this model. In Phase 2, the best performing model for the NO_2_-A43F sensor is the MR that includes the sensor reading, T, and RH using all data with a predictive r-squared of 0.81 (Table 1). All calibration results presented in Table 1 used a multivariable regression model except for the NO_2_-AE sensor during Phase 2, which used a polynomial regression.

**Figure 5:**
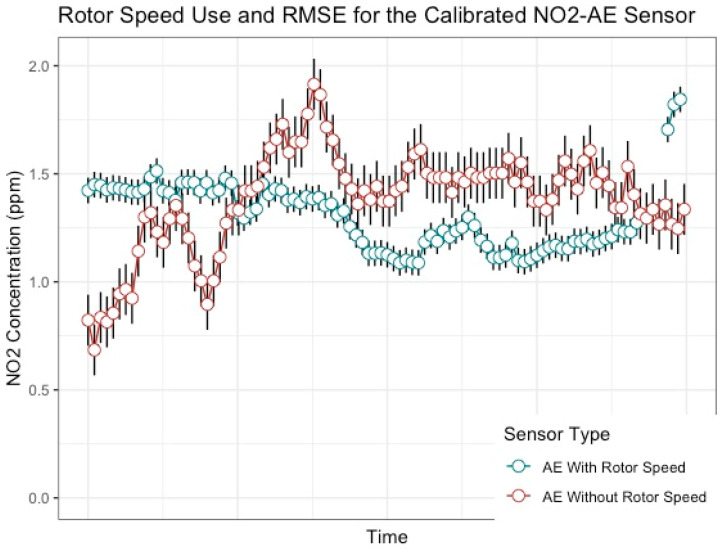
Comparing NO_2_ concentration for the calibrated NO_2_-AE sensor using the best model where rotor speed is used, and the second-best model, where rotor speed is not used. The RMSE is indicated as error bars for each data point.

In Stage A of Phase 3, the best location for calibrating each of the sensors while on board the UAV is the FOT for the NO_2_-A43F sensor and the TOC for the NO_2_-A43F sensor. These models have the lowest RMSE where rotor speed is a statistically significant addition to the model (Table 1). Removing rotor speed from these models results in a lower predictive r-squared. In Figure 5, a comparison is made between the NO_2_ concentrations for the calibrated NO_2_-AE sensor using the best model where rotor speed is used, and the second-best model, where rotor speed is not used. The RMSE is indicated as error bars for each point. The error bars associated to the model where rotor speed is used are smaller than the error bars associated to the model were rotor speed is not used, suggesting that the models without rotor speed have higher variability and uncertainty, and are therefore are not as reliable as models that do use rotor speed. Other models performed very poorly, such as the TOT sensor placement for the NO_2_-A43F sensor, which resulted in a very low predictive r-squared (Table 1).

**Figure 6:**
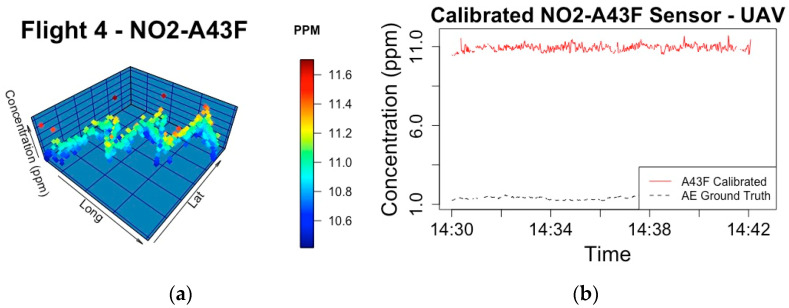
(**a**) Flight path and ppm concentration during Flight 4 of Stage B during Phase 3 of calibrating electrochemical sensors, where the NO_2_-A43F sensor is mounted on the UAV; (**b**) The calibrated sensor concentration is compared to the ground truth concentration.

**Figure 7:**
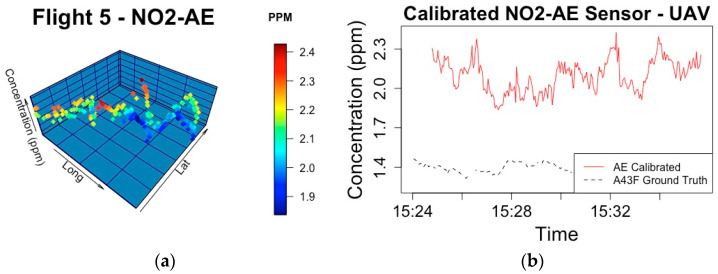
(**a**) Flight path and ppm concentration during Flight 5 of Stage B during Phase 3 of calibrating electrochemical sensors, where the NO_2_-AE sensor is mounted on the UAV; (**b**) The calibrated sensor concentration is compared to the ground truth concentration.

In Stage B of Phase 3, the ground truth concentrations, which are derived from the sensor attached to the rucksack as outlined in the research methodology, are calculated by the best performing models from Phase 2. Flights 1 and 2 tested different flight patterns to find the plume, and Flight 3 had problems processing the dataset. Each sensor setup and the associated RMSE of Flights 4 and 5, when the validation occurred, can be seen in Table 2. The RMSE error is attributed to the performance of the UAV calibration where rotor speed is included in the best performing model. Figure 6a and Figure 7a present the mapped plume during each of these flight experiments and the calibrated NO_2_ concentration in ppm. Figure 6b and Figure 7b presents a time series between the calibrated sensor and the associated ground truth measurements for validation.

## 4. Discussion

Previous studies have shown that the optimal location of a gas sensor while mounted on an UAV is for it to be as close to the gas being analyzed and as far away from the propellers as possible—suggesting the influence that rotor speed has on determining gas concentrations [7]. While studies have calibrated electrochemical sensors for such experiments, none have considered using variables like rotor speed as part of the calibration model to account for potential interference from UAVs [10,11,29]. As a response, this study has built on previous research by including rotor speed as an independent variable to account for interference from an UAV when calibrating electrochemical sensors for detecting NO_2_ [5].

Results show that this variable is a statistically significant addition to the final model, confirming the hypothesis that rotor speed from an UAV influences the overall concentration of NO_2_ and can be included in a calibration model to acquire more reliable concentrations of NO_2_ than models that do not include rotor speed (Table 1). Figure 5 is also representative in visualizing this by the smaller RMSE values for each data point. Calibrating electrochemical sensors with rotor speed can therefore support more accurate and reliable NO_2_ concentrations while further developing an understanding of the transport of gas to gas sensors in a way that can be advantageous for future work concerning the integration of UAV’s and electrochemical sensors [6,11,29].

This research provides some very useful insight into the potential of adding other variables, such as wind speed or air pressure, that might be interfering with the sensor reading when using an UAV to detect gases with electrochemical sensors and suggests that these models and calibration procedures should be further investigated in order to optimize results. For example, the NO_2_-A43F sensor has a much lower predictive r-squared than the NO_2_-AE sensor in Stage A, which might be attributed to error propagation associated with exogenous variables when calibrating the sensor for UAV experiments (Table 1). Such variables may have contributed to the gaps in concentrations seen between Flights 4 and 5 in Stage B.

Future Research

Acquired wind speed data was averaged hourly and would therefore not have been useful for electrochemical sensor calibration in these UAV experiments. Therefore, future experiments will have to address these issues where minute-by-minute wind data is readily available and follow procedures such as those conducted by Villa et al. with the best performing models presented in this research in order to have more control of the environment.

Furthermore, there should be at least more than one day dedicated to calibrating the sensors on board the UAV, where multiple flights can take place for calibrating each sensor. For optimal results, a calibration should take place before each flight. However, under most circumstances where gas chambers or official stations are not readily available, achieving this is not always feasible. Other experiments can test other locations around the emission source and use the denuder for siphoning desired gases, which might favor results if directly connected to the sensor as seen in Rüdiger et al. Sensing systems that retain target gases with chambers should also be considered [33].

Supplementary Materials in this research also provide raw and preprocessed data collected from the UAV and electrochemical sensors during each of the calibration phases, which future research can freely use and build from. Open source data in this research domain is scarce, so the authors are proud to offer this information as a free resource for the scientific community and any individuals hoping to expand this study.

Upon following these recommendations, other sensors or drones can be used in order to identify how well each of these models perform between different sensing designs. Machine learning techniques can also be used instead of regression models for calibration. Obtaining an accurate and reliable calibration of electrochemical sensors, and any other small, low-cost sensor, still remains a big challenge [5,7]. Bigi et al. explained that its complexity lies in the fact that since there is a multitude of sensing applications and each application requires specific tools and parameters, and that without concrete physical or chemical working principles set for each sensor, it would be difficult to meet optimal performances of all devices. In other words, calibrating a sensor is unique for every individual sensor and requires, at the very least, a slightly different calibration model for each individual sensing application. Despite these intricacies, it is likely that different algorithms will be generated as more experiments are conducted in order to account for more of these unique circumstances while lessening the need for complex calibration procedures. For now, UAV experiments can benefit by including variables like rotor speed in electrochemical sensor calibration models in order to derive more reliable sensor readings.

## 5. Conclusions

With the intent of expanding the spatial resolution of and capacity for detecting toxic gases, this research has utilized traditional and novel electrochemical sensor calibration procedures to obtain more realistic concentrations of NO_2_ when conducting UAV experiments. All three calibration phases used a series of regression models and a combination of exogenous variables known to interfere with electrochemical sensor readings, including rotor speed. Results indicate that these methods may provide more reliable concentrations of NO_2_ when rotor speed is included in the calibration model.

## Figures and Tables

**Table 1:** Calibration Results for Phase 1, 2, and Stage A of Phase 3.

Phase	Sensor	Ground Truth	Split/Sensor Placement	Date/Time	Model Components	Predictive R^2^	RMSE
1	NO_2_-AE	Amsterdam Vondelpark	3rd Quarter	30/11/18 07:00:00–02/12/18 08:00:00	Signal and RH	0.77	0.0021
2	NO_2_-AE	GGD Amsterdam	2nd Quarter	11/12/18 10:55:51–11/12/18 11:16:31	Signal^3^, T^2^, RH^2^	0.97	0.0099
2	NO_2_-A43F	GGD Amsterdam	All Data (no split)	11/12/18 10:35:00–11/12/18 12:00:00	Signal, T, RH	0.81	0.0030
3	NO_2_-AE	Calibrated NO_2_-AE	FOT	16/07/19 11:26:59–16/07/19 11:31:20	Signal	0.21	0.1962
3	NO_2_-AE	Calibrated NO_2_-AE	TOT	16/07/19 11:31:21–16/07/19 11:35:22	Signal, RH	0.75	0.1194
3	NO_2_-AE	Calibrated NO_2_-AE	TOC	16/07/19 11:35:23–16/07/19 11:43:20	Signal, T, Speed	0.80	0.0624
3	NO_2_-A43F	Calibrated NO_2_-A43F	FOT	16/07/19 16:09:03–16/07/19 16:12:16	Signal, T, RH, Speed	0.50	0.0507
3	NO_2_-A43F	Calibrated NO_2_-A43F	TOT	16/07/19 16:12:18–16/07/19 16:13:58	Signal	−0.04	0.1092
3	NO_2_-A43F	Calibrated NO_2_-A43F	TOC	16/07/19 16:14:01–16/07/19 16:17:45	Signal, T, RH	0.43	0.2217

This table represents the best performing models for the NO_2_-A43F and NO_2_-AE sensors during Phase 1, Phase 2, and Stage A of Phase 3.

**Table 2:** Stage B of Phase 3 Validation Results.

Flight Number	UAV Sensor	Ground Truth Sensor	RMSE
4	NO_2_-A43F	NO_2_-AE	9.5584
5	NO_2_-AE	NO_2_-A43F	0.7520

Validation results in Stage B of Phase 3, where the calibrated sensors are used as a ground truth for when the other sensors are on board an UAV.

## Supplementary Materials

In order to support future testing in these fields, the dataset from the NO_2_-A43F and NO_2_-AE electrochemical sensors and the UAV telemetry used in this research has been made publicly available via the following GitLab link https://git.wur.nl/said-lab/drone-olfaction-calibration.
